# Effect of COVID-19 lockdown on regional pollution in Ireland

**DOI:** 10.1007/s11869-021-01098-4

**Published:** 2021-09-28

**Authors:** Teresa K. Spohn, Damien Martin, Michael Geever, Colin O’Dowd

**Affiliations:** Centre for Climate and Air Pollution Studies (C-CAPS), School of Physics, National University of Ireland Galway, University Road, Galway, Ireland

**Keywords:** COVID-19 restrictions, Regional pollution, Ireland

## Abstract

This study examines the regional impact of the COVID-19 lockdown restrictions on pollution in Ireland by comparing the 2020 measurements of ozone (O_3_), nitrogen dioxide (NO_2_), and particulate matter (PM) from monitoring stations around the country to the previous 3-year average. Results indicate that O_3_ was 5.6% lower and 13.7% higher than previous years during the lockdown at rural and suburban sites, respectively. NO_2_ decreased by 50.7% in urban areas, but increased slightly in agricultural regions, consistent with satellite observations. PM concentrations did not change significantly compared to previous years; however, a reduction in the signal variability in the smaller size particle measurements may be the result of different emission sources. The reduction in NO_2_ likely increased the ratio of volatile organic compounds (VOCs) to NO_x_ (nitrogen oxides), creating a NO_x_ limited environment, which resulted in an initial increase in O_3_ in suburban areas, and the lower than usual levels observed at rural sites. Meteorology showed higher than average wind speeds prior to lockdown, which likely acted to disperse PM and NO_2_.

## Introduction

With the COVID-19 pandemic currently affecting the entire world, it has been observed in other places such as China, Italy, and the USA that pollution levels have dropped significantly as a result of less traffic and industry ([Fan et al. 2021; Altuwayjiri et al. 2021; Goldberg et al. 2020]). Already the Irish EPA has reported a 50% reduction in nitrogen dioxide (NO_2_), which is the primary pollutant emitted by road traffic, for Dublin after lockdown restrictions were put in place according to a recent press release [EPA-Ireland 2020].

While these changes are most visible in cities and highly industrial areas, as shown for example by Collivignarelli et al. (2021), who demonstrated that despite the lockdown restrictions large cities still exceeded NO_2_ limits, this paper will examine if changes on a regional scale are detectable in Ireland. In addition to NO_2_, particulate matter (PM) and ozone (O_3_) are investigated, as these both are related to NO_2_ emissions. In order to do this, measurements from the three coastal stations Mace Head, Malin Head, and Carnsore Point which form the newly established national Atmospheric Composition and Climate Change (AC^3^) network, in conjunction with available O_3_, PM10, and NO_2_ data (downloaded from the European Environmental Agency website [EEA 2020b]) from sixteen other sites across the country operated by the Irish Environmental Protection Agency will be analysed and compared to other studies. This includes five urban, nine suburban, and five rural stations.

Meteorology can have a significant impact on NO_2_, PM, and O_3_, as high wind speeds disperse concentrations, precipitation washes out particles, and solar radiation, temperature, and humidity influence the chemical production cycle of O_3_ from NO_2_. A recent study by Ordóñez et al. (2020) showed that higher than average temperatures and solar radiation led to increased levels of O3 across central Europe in 2020, despite varying degrees and time periods of lockdown restrictions. Due to a lack of relevant meteorological data at the locations chosen, in order to account for meteorology and seasonal cycles, the average of three previous years of data will be used to assess changes.

After the World Health Organization (WHO) declared the COVID-19 virus a global pandemic in early March 2020, most countries, including Ireland, began to enforce varying degrees of lockdown restrictions. Ireland’s response was first to close all schools and bars as of 16 March. Beginning on 28 March, a full lockdown came into effect with all non-essential businesses closed, and people working from home and limiting their movement to within 2 km of their homes wherever possible. This lasted until 19 May, when lighter restrictions allowed for certain businesses and restaurants to reopen, as well as a wider range allowed for travel. The changes resulting from the sharp decrease in traffic during the hard lockdown period will be examined here.

## Material and methods

### Stations and instrumentation

**Fig. 1:**
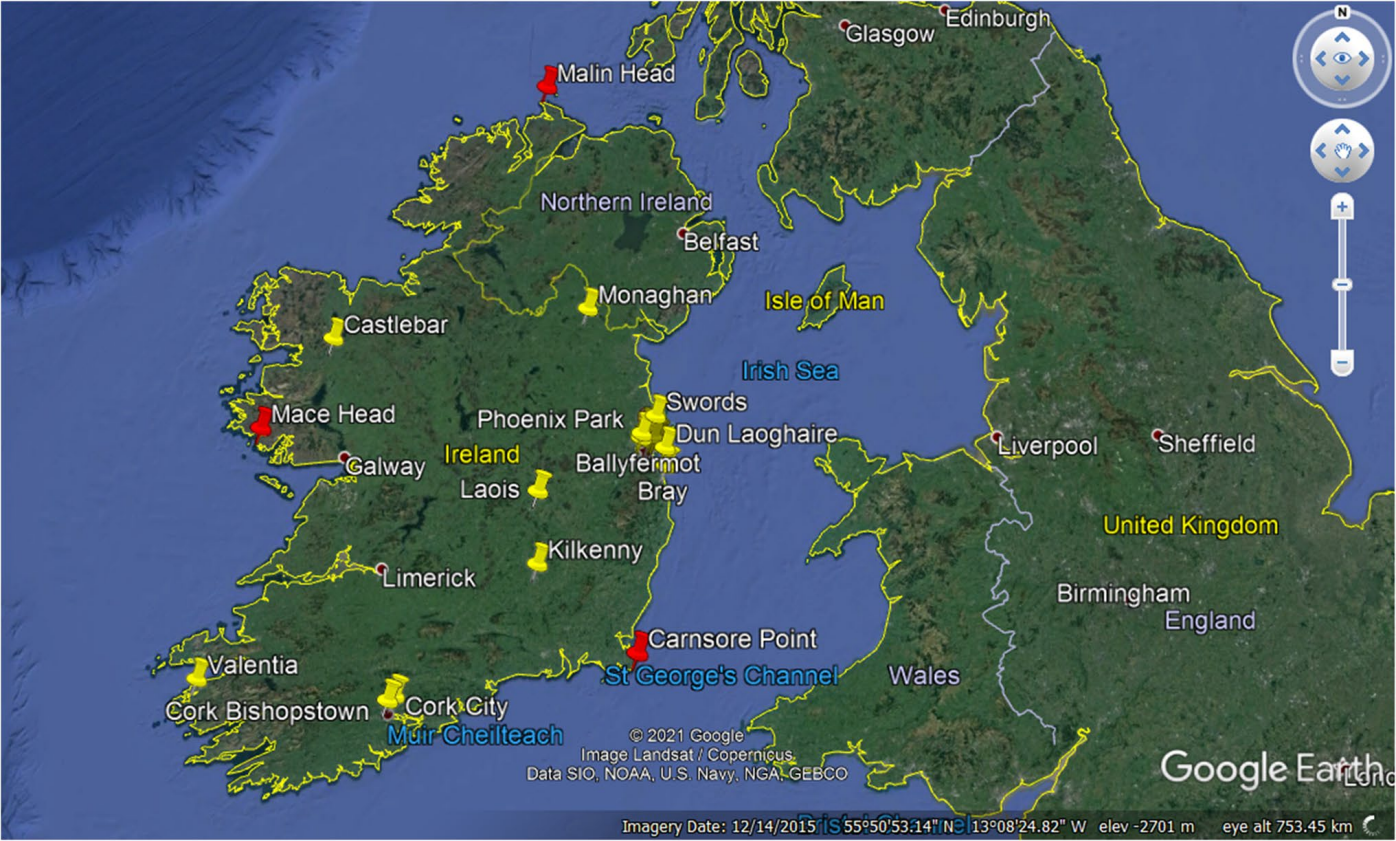
Irish EPA stations (yellow pins) AC^3^ Network (red pins), image courtesy of Google Maps

The stations, their locations, classifications, and measured species for this study are listed in Table 1. Although some of these stations measure other species as well, only datasets where sufficient data were available were used. The classifications denote distance from sources, mainly traffic, with urban sites being in closest proximity to heavy traffic, and rural sites being at least 10 km distant. The corresponding map in Fig. 1 provides a visual interpretation of the site locations, showing the majority of stations within the Dublin area.

**Table 1:** Stations used in this study

Station	Lat/Long	Class	O_3_	NO_2_	PM10
Cork City	51° 54′ 39.60″ N, 8° 28′ 30.00″ W	Urban	X	X	X
Finglas (Dublin)	53° 23′ 25.08″ N, 6° 18′ 18.72″ W	Urban		X	X
Rathmines (Dublin)	53° 19′ 14.40″ N, 6° 16′ 40.80″ W	Urban	X	X	
Ringsend (Dublin)	53° 20′ 30.48″ N, 6° 13′ 35.40″ W	Urban			X
Woodquay (Dublin)	53° 20′ 52.44″ N, 6° 14′ 37.68″ W	Urban			X
Ballyfermot	53° 20′ 24.72″ N, 6° 21′ 6.12″ W	Suburban		X	X
Bray	53° 11′ 13.20″ N, 6° 7′ 19.20″ W	Suburban	X		
Clonskeagh (Dublin)	53° 18′ 43.20″ N, 6° 14′ 6.00″ W	Suburban	X		
Cork Bishopstown	51° 53′ 2.40″ N, 8° 31′ 58.80″ W	Suburban	X	X	
Dun Laoghaire (Dublin)	53° 17′ 9.60″ N, 6° 7′ 55.20″ W	Suburban		X	
Kilkenny	52° 38′ 20.40″ N, 7° 16′ 4.80″ W	Suburban	X	X	
Laois	53° 2′ 9.60″ N, 7° 17′ 20.40″ W	Suburban	X	X	
Phoenix Park (Dublin)	53° 21′ 52.56″ N, 6° 20′ 53.16″ W	Suburban			X
Swords (Dublin)	53° 27′ 47.16″ N, 6° 13′ 19.92″ W	Suburban		X	
Carnsore Point	52° 10′ 14.16″ N, 6° 21′ 20.16″ W	Rural	X		
Mace Head	53° 19′ 28.92″ N, 9° 54′ 11.88″ W	Rural	X		
Malin Head	55° 22′ 55.20″ N, 7° 22′ 24.24″ W	Rural	X		X
Monaghan	54° 3′ 57.60″ N, 6° 52′ 58.80″ W	Rural	X	X	
Valentia	51° 56′ 16.80″ N, 10° 14′ 27.60″ W	Rural	X

Ozone measurements were conducted using Thermo Fisher Scientific O_3_ Analysers. These work by drawing the air into two sample chambers, one of which is scrubbed of ozone and acts as the reference gas, and the other which becomes the sample gas. These alternately flow through a solenoid valve and are exposed to ultraviolet light at 254 nm, and the Beer Lambert Law is applied to calculate the ozone concentration from the reference and sample gases [Thermo-Fisher-Scientific 2020].

Nitrogen dioxide concentrations were obtained indirectly from chemiluminescent NO_x_ (nitric oxide + nitrogen dioxide) monitors. This is done by photometrically measuring the light intensity at wavelengths greater than 600 nm which occurs when nitric oxide (NO) and ozone react. The process involves first converting the NO_2_ from one part of the sample to NO, representing the total NO_x_, and comparing it to another sample which has been allowed to react with ozone. The difference between the two samples is equivalent to the NO2 concentration [EPA-US 2002].

A Palas Fidas instrument was used for measuring particulate matter at the stations. It is an aerosol spectrometer capable of simultaneously distinguishing between PM1, PM2.5, PM4, and PM10 sized particles at a very high time resolution and, as the only instrument capable of this, is currently the European standard for fine dust monitoring. The instrument works by measuring the number of scattered light impulses produced as particles pass through an optically differentiated measurement volume illuminated by white light, and converts them to a mass by multiplying by a correlation factor corresponding to different sources of environmental aerosol and applying a separation curve for size distribution [Palas 2019].

Meteorological measurements were not available for all locations, so data from the nearest Met Eireann stations (Ballyhaise, Co. Cavan; Cork Airport, Co. Cork; Dublin Airport, Co. Dublin; Johnstown Castle, Co. Wexford; Mace Head, Co. Galway; Malin Head, Co. Donegal; and Oak Park, Co. Wicklow) covering the time period 2017–2020 for the months of January through May were evaluated. Solar data were limited to those from Dublin and Cork airports.

### Methodology

The method of data analysis employed was based on a recent study conducted in the United Kingdom by Lee et al. (2020), in which the combined previous 5 years’ average of the measurements was compared to 2020 before and after lockdown. However, as most sites in Ireland did not have data going back to 2015, only the average of the previous 3 years was used. Similar to the UK study, the Irish sites were classified into urban, suburban, and rural in order to observe how these different environments were affected. While the UK study focused on nitrogen dioxide (NO_2_) and ozone (O_3_), this study includes measurements of particulate matter (PM) as well. For the purpose of this study, the pre-lockdown period will be considered January and February, and lockdown will focus on the hard lockdown from 28 March to 19 May. In the case of ozone, the most visible changes occurred in May, and therefore only this month is used in comparing to the pre-lockdown period.

## Observations

### Nitrogen dioxide

**Fig. 2:**
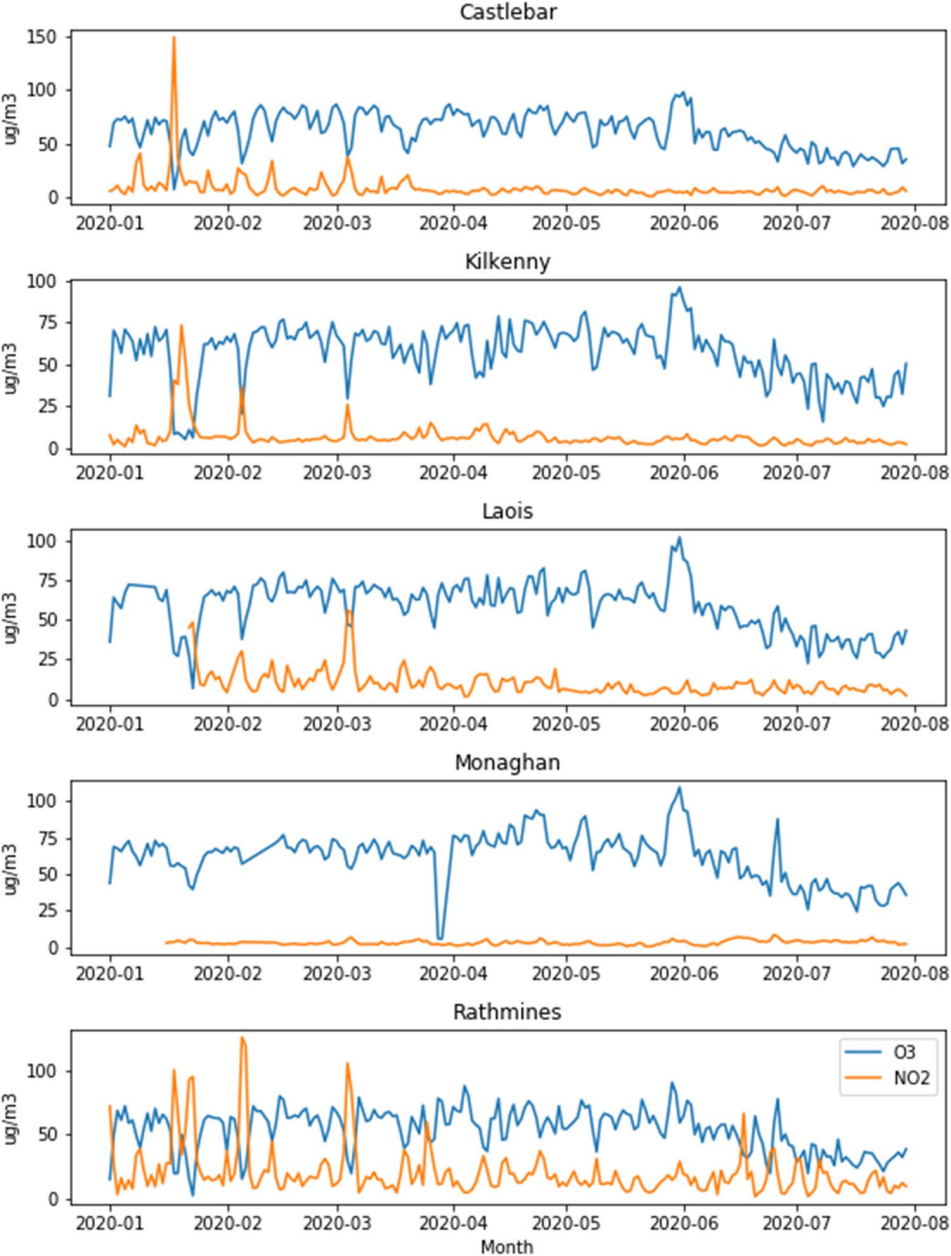
NO_2_ and ozone daily averages comparison

Nitrogen dioxide (NO_2_) is primarily emitted by vehicles, and therefore a key indicator of traffic-related changes in pollution. It is measured at five of the sites that also measure ozone, only one of which is rural. The comparison with ozone in Fig. 2 shows that the rural site (Monaghan) has significantly lower NO_2_ levels than the other stations, although its ozone levels are about the same as the others. Monaghan differed from the other rural sites in that it experienced a 1.88% increase in ozone during the lockdown period. The reduction in NO_2_ as a result of lockdown is clearly visible in Fig. 5, especially in the urban area of Rathmines, which likely experienced the greatest decrease in vehicle emissions.

**Fig. 3:**
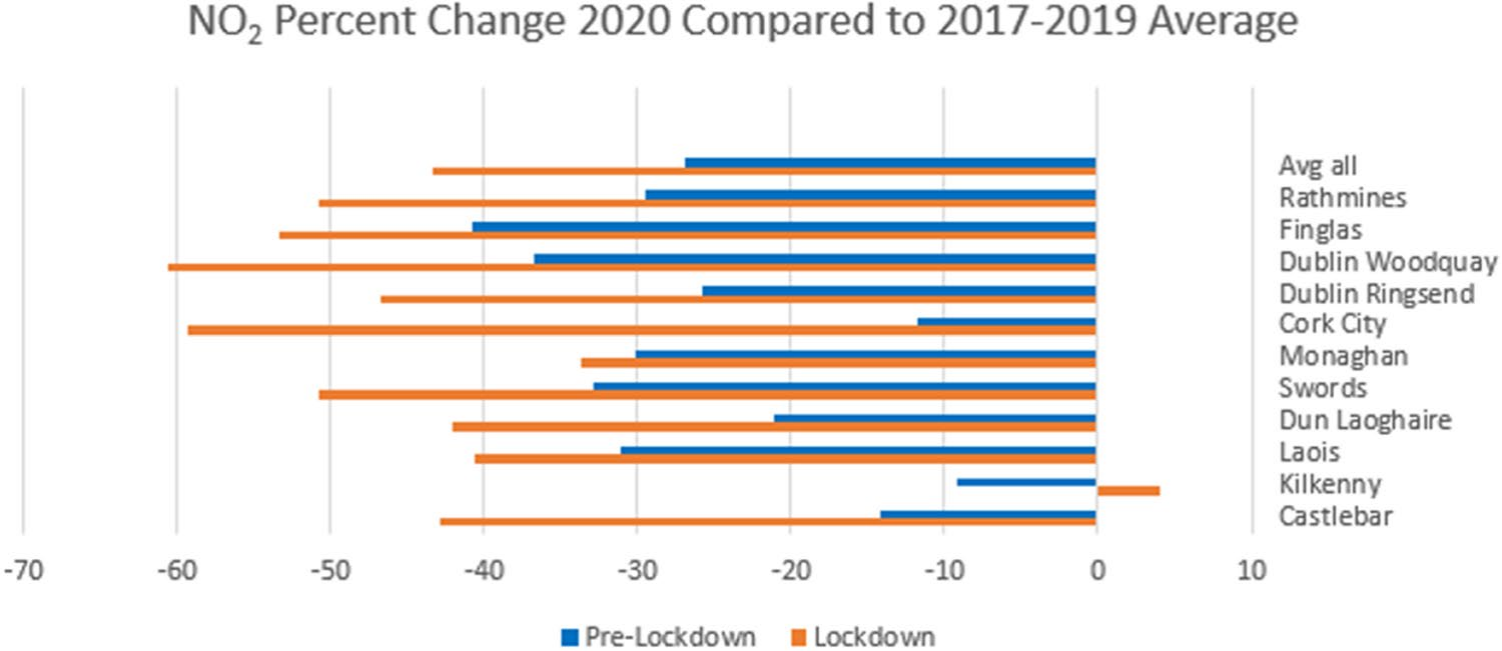
Percent change in NO_2_ between 2020 and the average 2017–2019

**Fig. 4:**
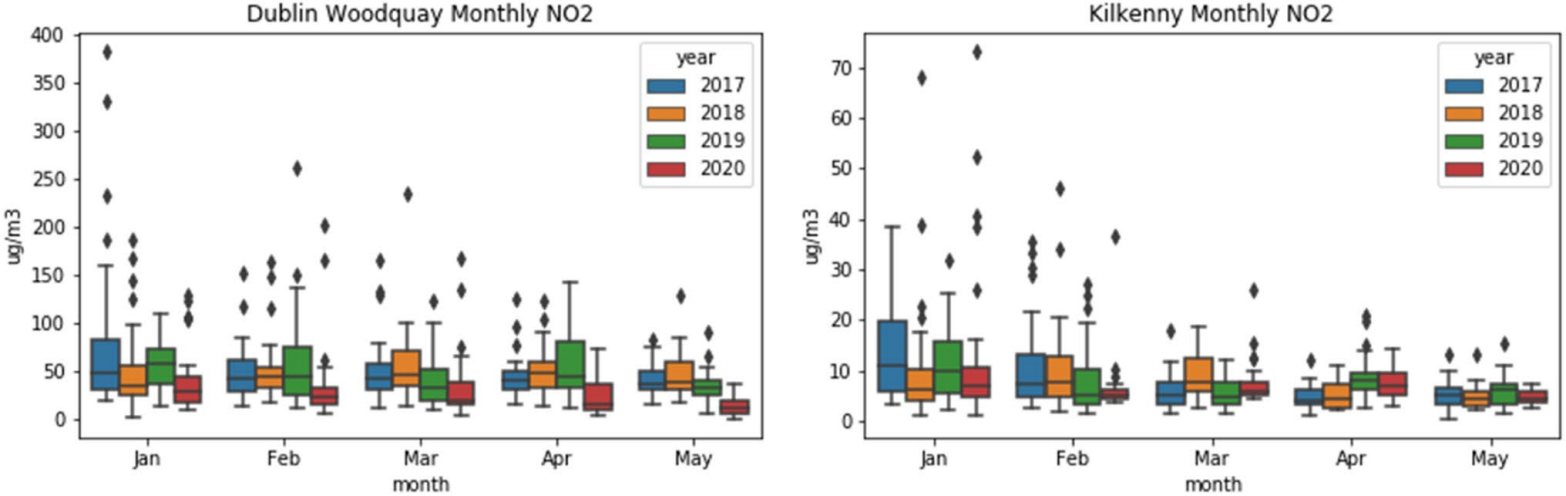
NO_2_ boxplots for Dublin Woodquay and Kilkenny

**Fig. 5:**
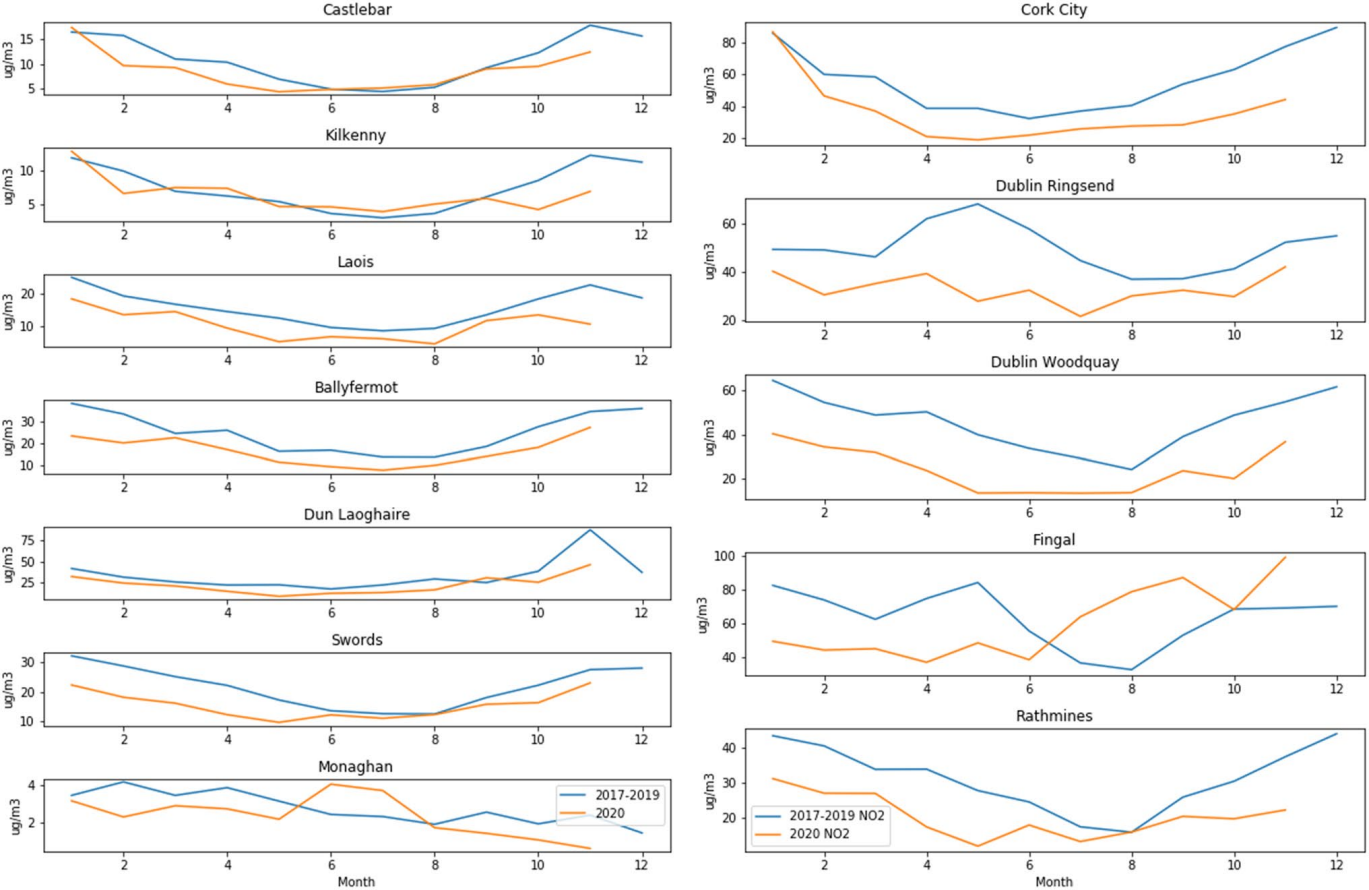
Suburban background plus rural Monaghan (left) and urban (right) NO_2_ comparison 2020 to average of 2017–2019

There are five Dublin sites measuring NO_2_ (but not O_3_), which were included here for comparison. Dun Laoghaire and Swords are background suburban sites, whereas Dublin Ringsend, Woodquay, and Finglas are categorized as urban traffic. As Fig. 3 shows, all stations had lower levels of NO_2_ in 2020 than in previous years; however, the difference increased everywhere except Kilkenny during the lockdown period. The heaviest traffic area of Dublin Woodquay is associated with the largest decrease (60.6%) compared to the previous years’ average during the lockdown period, and the average decrease over all Dublin sites was 50.7%, consistent with the EPA’s report [EPA-Ireland 2020]. NO_2_ concentration depends on emissions, chemistry, and meteorology, and the largest source of emissions, traffic, had not changed compared to previous years before lockdown, meaning that the reduction is likely due to changes in meteorology, which will be looked at in more detail later. The boxplots in Fig. 4 for Dublin Woodquay and Kilkenny indicate that there was no visible upward or downward trend in previous years, and this holds true for all sites. The plots in Fig. 5 reflect this as well, with Kilkenny being the only station to show a net increase in NO_2_ during the lockdown. The reason for Kilkenny exhibiting a different pattern than the other stations is likely local or regional influences such as agricultural activity.

Satellite images from NASA’s Aura/OMI [NASA 2020] during the lockdown period between 28 March and 19 May 2020 also show reductions in NO_2_ compared to previous years of about 27% (± 13% over the time period) overall in Dublin This is less than the average decrease in NO_2_ of 50.7% (± 6.2% of the site averages) measured in situ across all the Dublin sites, likely due to the satellite comparison covering a larger time scale for previous years (2015–2019), and comparison of a point measurement to a spatial footprint. The satellite analysis is focused on Dublin; however, the maps do show the entire country. Here it is noteworthy that areas such as Kilkenny seem to have slightly higher than usual NO_2_ concentrations, which is consistent with in situ measurements from that station. According to Duffy et al. (2020), transport made up 40.6% of the national total NO_x_ in Ireland, but agriculture accounted for 32.4% of NO_x_ emissions. As Kilkenny is located in a largely agricultural part of Ireland, it is likely that the 4% increase in NO2 compared to previous years is related to that.

### Ozone

Ozone is a harmful secondary pollutant formed by photolysis of vehicular emissions of nitric oxide (NO) and nitrogen dioxide (NO_2_), collectively known as NO_x_, and volatile organic compounds (VOCs), and so may be affected by a reduction in these pollutants. It is being measured at the rural sites Mace Head, Malin Head, Carnsore Point, Valentia, and Monaghan, as well as suburban background sites of Bray, Castlebar, Cork Bishopstown, Kilkenny, Laois, and Clonskeagh (Dublin). Cork City and Rathmines (Dublin) are classed as urban traffic sites.

**Fig. 6:**
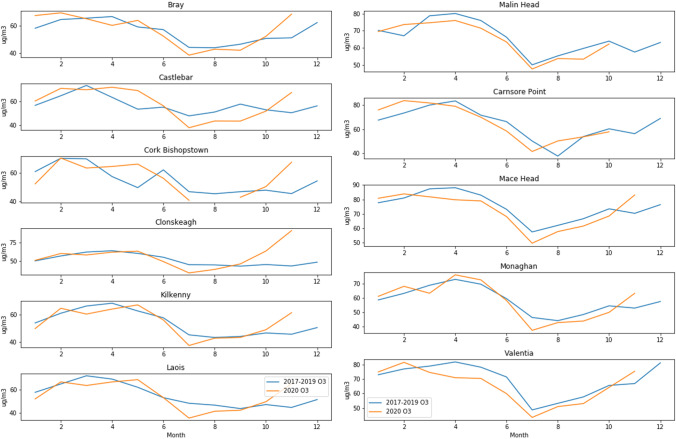
Suburban background (left) and rural ozone (right)

**Fig. 7:**
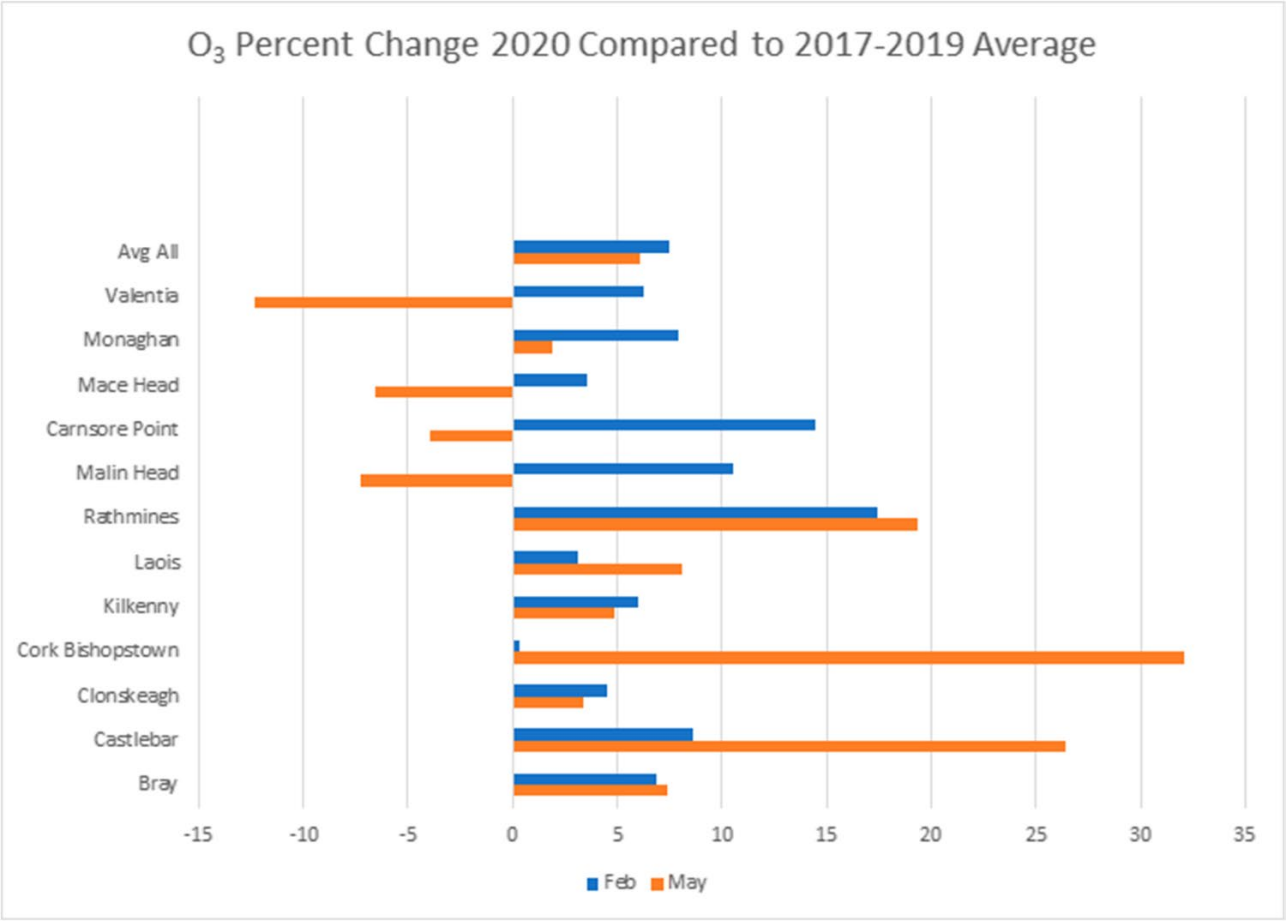
Percent change in ozone between 2020 and average of 2017–2019

**Fig. 8:**
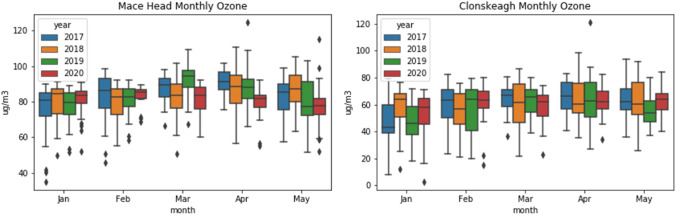
Comparison of rural (Mace Head) and suburban (Clonskeagh) ozone

Looking at the differences between 2020 ozone concentrations and the previous 3-year average, some interesting patterns emerge. As shown Fig. 6, areas considered suburban background experienced a slight increase in ozone occurs during lockdown in May, on average 13.7%, whereas in rural areas there is an average 5.6% decrease in ozone during the same time period. The two urban traffic sites in Cork City and Rathmines (Dublin) showed no clear pattern relating to lockdown. By comparison, ozone was slightly higher than previous years in February (before lockdown), an average of 7.46% across all sites (Fig. 7). The boxplots in Fig. 8 illustrate typical examples of rural and suburban sites and show that there is no obvious upward or downward trend in ozone over the years.

It is noteworthy that at both suburban background and rural sites, the average monthly ozone is lower than the previous years’ average for the entire period after lockdown, with sharp increases toward the beginning of October when another lockdown came into effect. Plotting the ozone percent change by latitude showed no obvious relationship and is therefore not shown here. While meteorology plays an important role in ozone formation, the main factor influencing whether or not ozone increased or decreased at a particular site appears in this case to be its proximity to traffic, and hence NO_x_ emissions.

One possible reason for the sudden increase in ozone in the suburban areas could be what has become known as the “weekend effect”, described by Atkinson-Palombo et al. (2006) and references therein. This, as the name suggests, mainly occurs on weekends in suburban areas, when ozone increases as a result of fewer NO_x_ emissions, and possibly more sunlight from lower BC and PM levels, associated with traffic. Especially the amount of ozone titration through NO would have an effect if the NO_x_ concentrations are reduced. This could explain why in high traffic areas (which still experience higher volumes of traffic than suburban areas, even during lockdown), fresh NO emissions help to remove ozone, whereas suburban sites no longer have sufficiently high concentrations of NO. A recent study [Sicard et al. 2020] showed that the weekend effect could actually have been amplified by the lockdown measures in certain large cities.

As ozone formation in rural areas is more dependent on VOC concentrations than on NO_x_, the reduction in ozone is more difficult to explain. As discussed by Kroll et al. (2020), there is a complex and non-linear chemistry involved, which depends on the types of VOCs and their ratio to NO_x_, that determines whether ozone or secondary organic aerosol (SOA) is formed. Without more detailed measurements of VOCs, it is impossible to determine if the amount was reduced or increased due to anthropogenic emissions (less traffic, more cleaning agents), or if this affected the ratio to NO_x_ in such a way that SOA formation increased.

### Particulate matter

**Fig. 9:**
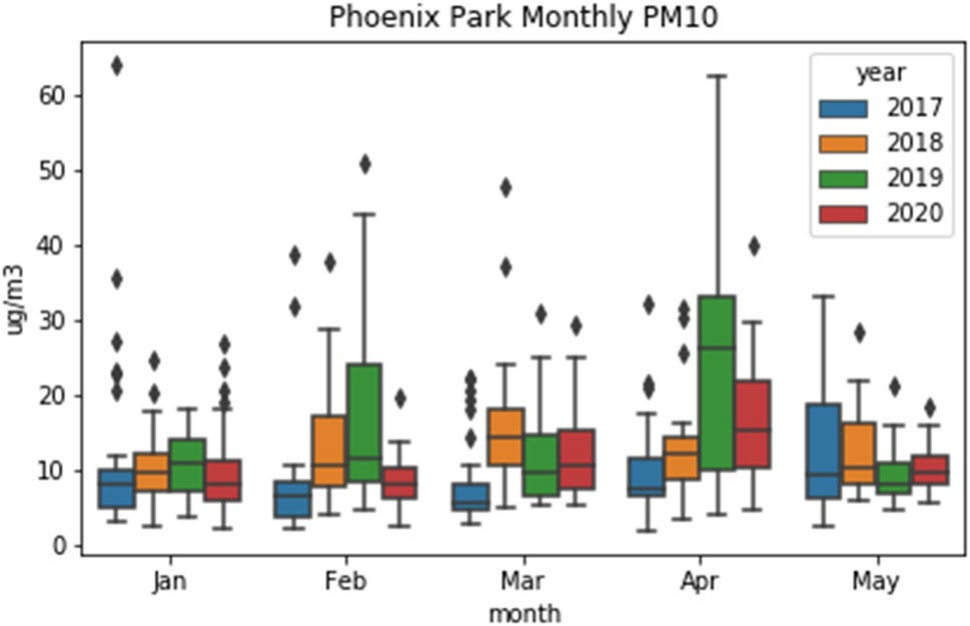
Boxplot of PM10 at suburban site Phoenix Park (Dublin)

Although most of the sites do have some available PM10 measurements, only seven of them had consistent enough data sets to complete this analysis. These were Dublin Ringsend, Rathmines, Ballyfermot, Phoenix Park, Cork City, and Castlebar. Only four sites measure PM2.5, all of them urban traffic apart from Malin Head, yet due to insufficient data an analysis could not be conducted. When comparing the 2020 PM10 measurements from the stations to the average of 2017–2019, PM10 did not change considerably before or during lockdown, and there was no consistent pattern in the percent change. This is likely due to the complex nature of PM, which has many sources and varied composition. The boxplots in Fig. 9 show only April 2019 being anomalously high compared to other years, which is reflected across all sites. As shown in a presentation by Coleman et al. (2020), wind speeds in the months of January and February were higher than the 2016–2019 average for those months, and may have caused dispersion of PM leading to lower concentrations in most areas, whereas there was less precipitation in the months after lockdown began which could mean fewer particles were washed out. Castlebar on the west coast of Ireland may have experienced increased sea salt as a result of the higher wind speeds. The composition of aerosols will vary by season and type of site, i.e. Malin Head PM10 is largely made up of sea salt, whereas Dublin would have more traffic and industry-related PM, but other factors such as an increase in home heating and decrease in road traffic due to lockdown could have resulted in similar levels of PM from different sources than usual.

**Fig. 10:**
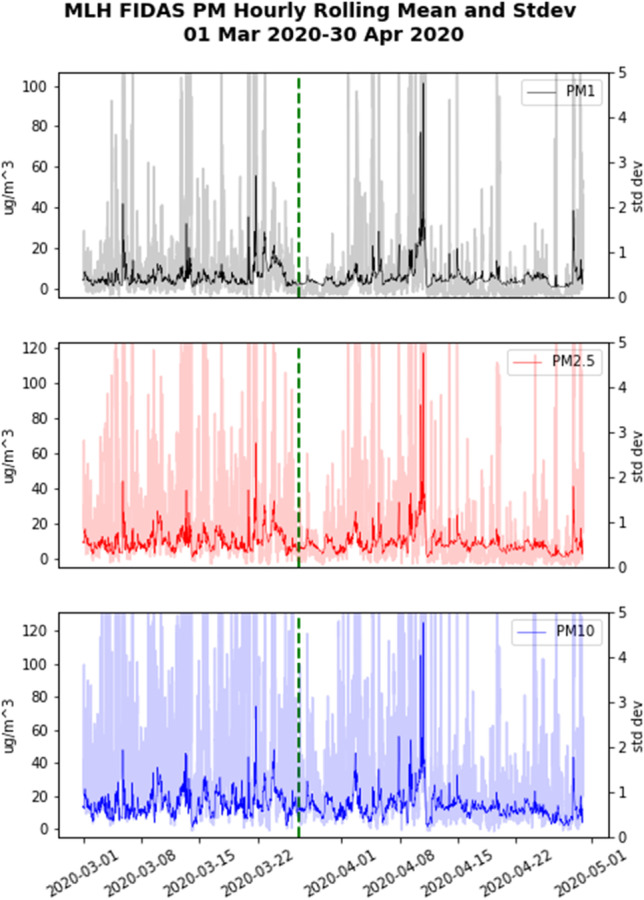
MLH Fidas PM hourly rolling time series on left axis with standard deviation in lighter colour on right axis (green dashed line denotes beginning of lockdown in Ireland)

As the Malin Head Fidas instrument has the ability to measure different PM size categories simultaneously, these data were further evaluated, and showed there were some clear changes after the lockdown comes into effect in Ireland. The time series in Fig. 10 shows a distinct reduction in the signal variability of PM1 and PM2.5 after lockdown begins (the average standard deviation for PM1 before lockdown was 1.00 and after lockdown 0.36, for PM2.5 before lockdown 1.60 and after lockdown 0.57), which could be related to less local traffic that would otherwise produce BC and road dust in the area. PM10 by comparison seems relatively unchanged with an average standard deviation of 1.05 before and 1.08 after lockdown, and with Malin Head being a coastal station, the sea salt component of these coarse particles would not be expected to change.

### Meteorology

Meteorology can affect all of the above species, so this was evaluated compared to previous years as well, using data available online from Met Eireann (the Irish meteorology service at https://www.met.ie) from the stations nearest the monitoring sites. As most of these sites are within Dublin, the main focus was on the Dublin airport data, which also provided information about hourly sun.

**Fig. 11:**
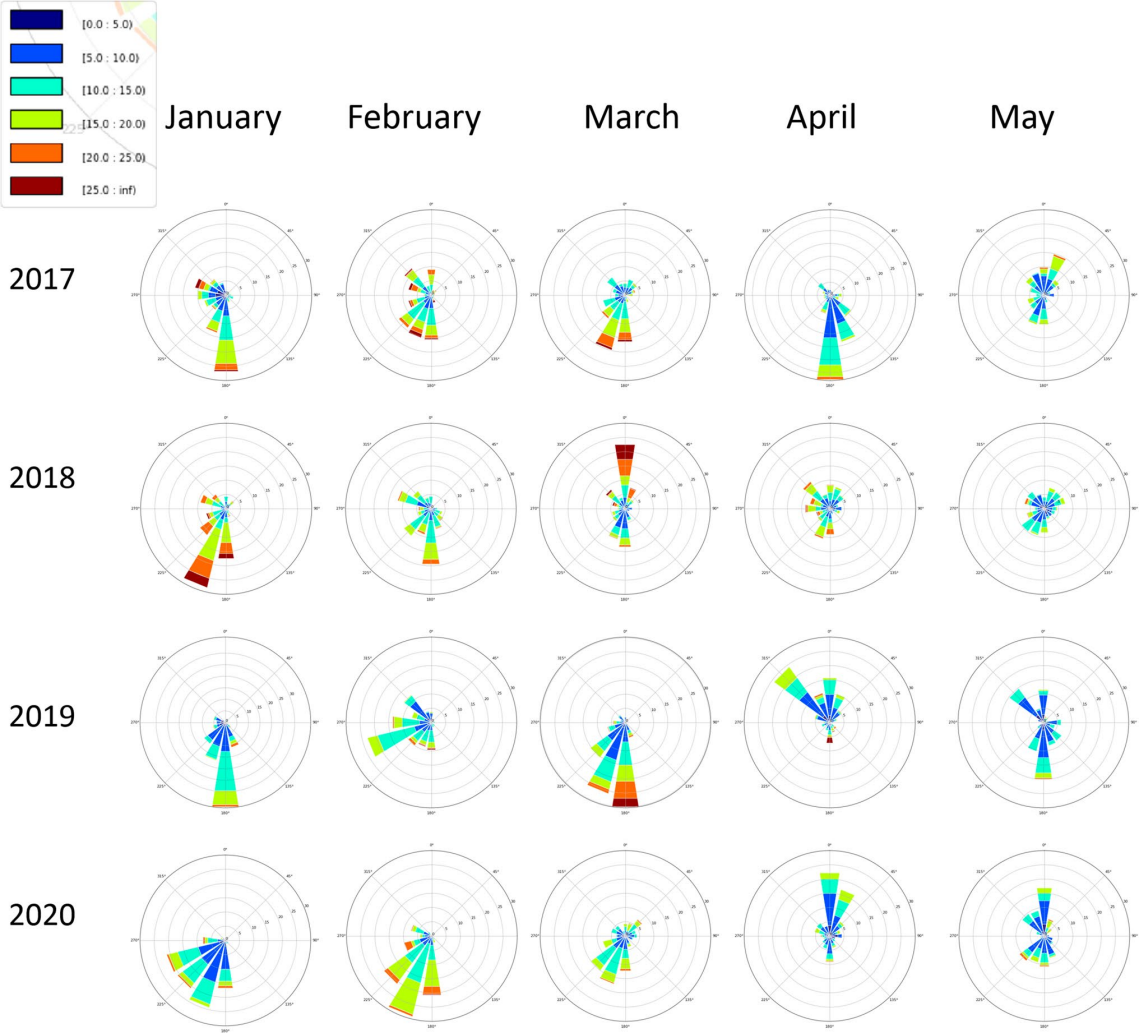
Dublin airport wind roses, scale is 0–30 knots for speed, colours represent frequency and occurrence

The most noteworthy changes in 2020 were the aforementioned windspeed and precipitation, although the wind roses also show a slight shift in wind direction to the southwest in January and February, which may have had the effect of bringing more Atlantic background air to Dublin than continental air from the south (Fig. 11). Higher wind speed would lead to greater dispersion, not only of PM, but also NO_2_, and could be a factor in the lower NO_2_ concentrations before lockdown.

The recent study by Ordóñez et al. (2020) evaluated the impact of meteorology on ozone production across Europe by modelling the expected concentrations during lockdown based on climatological data from 1981 to 2010. In this way, they were able to quantify how much of the change in ozone could be attributed to various factors such as temperature, solar radiation, humidity, wind speed, and precipitation. Although these often act in combination, i.e. higher temperatures are often the result of increased solar radiation and less precipitation, it was possible to isolate the main cause of the changes in ozone, which varied by region.

**Fig. 12:**
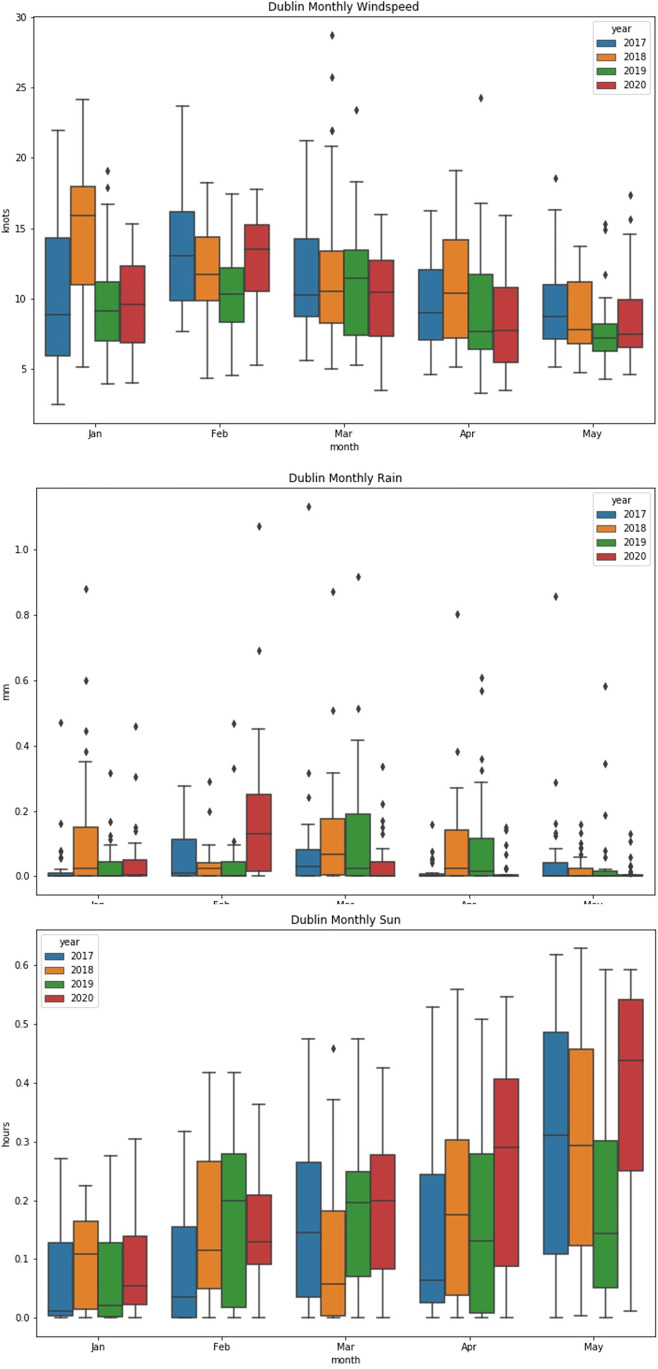
Dublin airport meteorology

As the study by Ordóñez et al. (2020) showed, the greatest influence on ozone production during lockdown in central Europe was solar radiation. In Dublin, solar radiation remained similar to previous years before lockdown, but was somewhat higher in April and May of 2020, which could affect ozone chemistry (Fig. 12). The maps for the March–April timeframe in the Ordonez study also seem to show this across the entire country, showing standardised anomolies in 2020 of a 0.1–0.5 increase compared to the same period in 1981–2010, but still lower than other parts of Europe, for example Germany, where this increase was greater than 1.0 .

More sunlight (and less precipitation) may also lead to higher temperatures, and as Ordonez et al. (2020) note, the main factor influencing the UK’s ozone production during lockdown appears to be higher than usual temperature, leading to an observed increase in ozone of 12.3 ± 13.9 percent in urban areas, and 3.1 ± 6.7 percent in rural areas compared to 2015–2019, vs. the climatological model which predicts only a 4.7 ± 11.4 percent increase in urban, and actually a 1.2 ± 5.5 percent decrease in rural areas. Indeed, their maps show a standardised anomaly of a 1.0–1.5 increase compared to previous years over the south and central part of England, with a south to north decreasing temperature gradient. This is similar to that found by Lee et al. (2020) where London, Bristol, and Cardiff experienced an average increase of 2C compared to previous years, whereas Glasgow and Belfast did not exhibit significant changes in temperature. The pattern is likely the result of less daylight with increasing latitudes at that time of year.

Looking at Ireland, the standardised anomaly of the temperature difference shown on the maps by Ordóñez et al. (2020) is at most 0.5, and therefore, the impact on ozone production is probably less than in the UK. Temperature data from seven Met Eireann stations across Ireland showed that all except Malin Head (the northern-most station) experienced a statistically significant increase in 2020 compared to 2017–2019, between 0.48C in Dublin and 1.16C in Oak Park (nearest station to Kilkenny), but only in April. The increases in ozone concentration in suburban areas occurred in May, and might be more related to traffic emissions, as other studies [Pusede and Cohen 2012] have shown that these tend to have a greater impact on ozone in cities than meteorology. In Kilkenny, the increased temperature could have played a role in the VOC and NO_x_ chemistry associated with both agricultural and seasonal emissions, and thereby led to an increase in NO_2_ in that region. While it is likely that meteorology did impact ozone production in Ireland, it is not possible to quantify how much with the data available.

## Analysis

### Comparison to literature

To put all of these results into perspective, it is informative to look at related measurements and studies. While a plethora of new literature showing reduced emissions in various parts of the world has emerged in the wake of the pandemic, here the focus will be on two articles which are most relevant to the observations in Ireland.

A recent study by Lee et al. (2020) examined in situ measurements at over 150 monitoring stations across the UK. Using the same methodology applied here of comparing 2020 values to previous years, they were able to show an average 11% rise in ozone in urban background areas of the UK after lockdown came into effect. They also found a slight increasing trend in ozone over multiple years. Solar radiation plays a major role in ozone formation, and the UK study demonstrated that the southern regions which had more sunlight and warmer temperatures during this period experienced greater increases in ozone compared to Scotland and Northern Ireland.

The increase in ozone in the suburban background sites of 13.7% in Ireland agrees well with the UK study by Lee et al. 2020; however, no upward trend could be discerned over time, with the more rural stations actually trending slightly downward. There was also no correlation between latitude and ozone concentration. These differences are likely due to fewer stations in Ireland with a shorter timeframe of measurements.

Menut et al. (2020) performed a modelling study comparing “business as usual” emissions to estimated reduced emissions based on how many fewer vehicles were on the road, and calculated that for Ireland this would result in a 2.7% reduction in ozone in urban areas, and a 2.34% reduction in ozone in rural areas. They also predicted a 37.3% and 29.5% decrease in NO_2_ in urban and rural areas, respectively. PM2.5 was expected to be 11.1% (urban) and 11.7% (rural) lower in Ireland.

Comparing the results from that study to the observations in Ireland, the average rural decrease of 5.6% in ozone is slightly higher than predicted for Ireland by Menut et al., yet close enough to be within the margin of error. While there was a short-term increase in suburban background ozone in May after the lockdown began, for the most part ozone did decrease in Ireland both in rural and suburban areas compared to previous years, though the percentage varies depending on the site’s proximity to traffic. The NO_2_ predictions of the reduced emission modelling scenario match very closely with the observations for both rural and urban environments; however, the PM2.5 did not reduce as predicted, indicating some other source of PM not related to traffic.

### Comparison to emission inventory

While NO_2_ was greatly reduced in most places as a result of less vehicular traffic during lockdown, O_3_ appears to have remained fairly constant before and after lockdown, as the daily averages show it to be well within the range of northern hemisphere background concentrations reported by Vingarzan (2004) of 20–45 ppb at all stations, even accounting for the springtime high. Apart from a brief increase in suburban areas compared to previous years from a possible “weekend effect” after lockdown, O_3_ was generally lower in 2020 than before. Ozone concentration depends on a number of factors, with both natural and anthropogenic sources. Its longer lifetime of about 20 days in the troposphere allows it to be transported over long distances, which is why there is a fairly constant background level.

The production and loss of ozone depends mainly on meteorology and the ratio of VOC/NO_x_. This relationship is often depicted in the form of an ozone isopleth diagram, as explained by Division on Earth and Life et al. (2000), with NO_x_ on the *y*-axis and VOCs on the *x*-axis, and lines of constant value of ozone (isopleths) plotted as the maximum resulting from the different combinations of NO_x_ and VOCs. A diagonal line from the origin at bottom left to top right on these charts is representative of the 8:1 ratio of VOC/NO_x_, and anything to the right of this line is considered NO_x_ limited (characteristic of rural or suburban areas, where a reduction in NO_x_ results in lower ozone), whereas to the left is VOC limited (in highly polluted inner cities, where in order to reduce ozone, VOCs must be reduced). Due to the curvature of the isopleths, it is possible in a VOC-limited environment for ozone to actually increase when NO_x_ is lowered, until the ratio crosses to the other side of the ridge line which results in reduced ozone. This also is part of the “weekend effect” in urban areas.

VOCs are a broad category of chemical compounds, many of which come from natural sources such as trees. Without measurements of these compounds, it is impossible to know how the concentrations and composition have changed, thus affecting the VOC/NO_x_ ratio. The Informative Inventory Report for Ireland [Duffy et al. 2020] estimates only 4% of total VOCs are related to transport. This is a very small amount compared to the over 40% of NO_x_ attributed to transport, and so it is likely that the reduction in NO_2_ would result in a higher VOC/NO_x_ ratio after lockdown, creating a more NO_x_ limited environment which would account for the somewhat lower O_3_ levels in 2020.

The MapEire.dk [MapEire.dk 2018] website depicts the Irish emissions inventory according to major sources by species on a spatial resolution of 1 × 1 km. The maps on this website show NO_x_ and VOC emissions from 2015 across Ireland. As NO_x_ is heavily associated with transport, roads and urban areas stand out. VOCs are largely associated with agriculture and industry, covering rural areas of the country, with increases in urban areas related to road use and solvents. Comparing the colour scales for these maps shows that VOCs are considerably higher than NO_x_, even in urban areas. Although these are only estimates, and the inventory is from 2015, this gives the impression that Ireland, with the exception of some very highly trafficked areas, is NO_x_ limited, and would explain why the model study by Menut et al. 2020 predicted a decrease in ozone for urban areas.

The European Environmental Agency (EEA) has created a tracker system online [EEA 2020a] where the levels of PM2.5 and PM10 can be observed on a weekly and monthly basis by country and city. On this website, they note that PM2.5 has not visibly reduced across much of Europe, due to its more varied sources, including agricultural ammonia, which would not have been reduced due to COVID-19 restrictions. Home heating had also not significantly declined during this period, according to the Irish EPA, likely due to people spending more time in their homes. As the most recent Informative Inventory Report for Ireland [Duffy et al., 2020] shows, transport made up only 8.7% of the total PM10 in 2018, and 13.8% of PM2.5, making it unlikely that a reduction in traffic would have a significant impact on these measurements.

Indeed, the Irish measurements of PM10 and PM2.5 do not appear to be greatly impacted by the lockdown. The percent increase/decrease was within a margin of error both at rural and urban sites. Even so, small changes were observed in PM1 at the rural sites. These could be related to other sources, i.e. what would normally have been traffic related might now be the result of increased home heating. The composition of PM is also very site dependent, as the coastal sites would be heavily influenced by sea salt.

### Impact of traffic

The Traffic Infrastructure Ireland (TII) website [TII 2020] tracks the number and type of vehicles for thousands of locations across Ireland. When comparing various sites before and after lockdown, there is a roughly 50–60% reduction in the daily amount of traffic, mainly related to passenger vehicles. For the Dublin site nearest to Rathmines, where previously a daily maximum of 900 vehicles were counted, after lockdown there were around 300 vehicles during the same late afternoon peak between 15:00 and 17:00. A rural area such as Monaghan that previously had about 350 vehicles at the peak afternoon traffic went down to 170 during the same period after lockdown. The level of NO_2_ reduction is directly linked to the amount of traffic, due to its shorter lifetime and higher concentrations closer to the source. Other TII sites around Dublin are located on very busy motorways, such as the M50, where there were over 10,000 vehicles/hour prior to lockdown, specifically on the section leading to the airport, and traffic was reduced to just over 3000 vehicles during the same time of day after lockdown. Monaghan on the other hand does not appear to be influenced by traffic emissions, as seen in the time series plot comparing O_3_ and NO_2_ at the stations. Of all the rural stations, it was the only one to show a slight (1.88%) net increase in O_3_ during lockdown, small enough and within the margin of error to be considered no change, and NO_2_ remained constant throughout.

Further analysis of the data showed that the Dublin site, in addition to experiencing a 60% reduction in passenger vehicles, also had the same decrease in light goods vehicles, buses, and heavy goods vehicles. Monaghan had a 50% decrease in passenger vehicles, yet the number of LGVs, buses, and HGVs did not decrease. As these types of vehicles tend to emit more pollutants, this is a possible reason that there is no visible change in NO_2_ levels measured at that station after lockdown.

## Conclusions

In summary, the COVID-19 lockdown did have a noticeable effect on regional pollution levels in Ireland. Ozone at rural sites was on average 5.6% lower compared to the previous years’ average, whereas it showed a 13.7% increase in suburban areas likely due to the “weekend effect”. These results agree with other studies which either showed or predicted similar changes. Nitrogen dioxide decreased at all stations relative to previous years, with the highest trafficked site in Woodquay (Dublin) associated with the greatest decrease of 50.7%. The only exception was Kilkenny, which is located in an agricultural part of the country which experienced higher than usual temperatures, and the increase in NO_2_ there is confirmed also by satellite imagery. Particulate matter did not appear to change considerably during this time, although it is possible the composition of particles may be different than usual, as the combustion sources changed from traffic to residential.

The methods used in this study carry a lot of uncertainty; however, they do give a good general impression of the impact of lockdown on Ireland. Future work may include a more complex analysis of the data combined with air mass trajectories which more clearly show regional transport, as well as further investigation into the effects of meteorology.

## Data Availability

Data were obtained from publicly available online sources provided by the Irish Environmental Protection Agency, in conjunction with measurements made by the National University of Ireland Galway.

## Code availability

Data processing was conducted using Python and Microsoft Excel applications.
